# Motivational interviewing training experiences among psychiatry and family medicine resident physicians: qualitative exploration in Oman

**DOI:** 10.1192/bji.2025.10053

**Published:** 2026-02

**Authors:** Yamamh Al-Jubori, Tamadhir Al-Mahrouqi, Kamila Al-Alawi, Raghad Almojahed, Rawan Albusaidi, Nazik Tayfor Babiker Ahmed, Mohammed Al Alawi

**Affiliations:** 1 Medical student, College of Medicine and Health Sciences, Sultan Qaboos University, Muscat, Oman; 2 Psychiatrist, Department of Behavioral Medicine, College of Medicine and Health Sciences, Sultan Qaboos University, Muscat, Oman; 3 Consultant, Medical Education Department, Oman Medical Specialty Board, Muscat, Oman; 4 Consultant, Department of Behavioral Medicine, College of Medicine and Health Sciences, Sultan Qaboos University, Muscat, Oman

**Keywords:** Medical education, motivational interviewing, psychiatry education, family medicine education, clinical competence

## Abstract

**Background:**

Motivational interviewing is a patient-centred communication approach designed to facilitate behavioural change by enhancing intrinsic motivation. Despite its widespread global utility, research on the training and applications of motivational interviewing among resident physicians in Oman remains untapped.

**Aims:**

To examine the awareness, training experiences and clinical implementation of motivational interviewing among psychiatry and family medicine residents enrolled with the Oman Medical Specialty Board (OMSB).

**Method:**

A qualitative study was conducted using semi-structured interviews and focus group discussions with 22 resident physicians from psychiatry and family medicine programmes. Data were analysed using thematic analysis to identify key themes regarding motivational interviewing training and its application in clinical settings.

**Results:**

Three primary themes emerged: (a) residents’ understanding and application of motivational interviewing principles, (b) barriers to the integration of motivational interviewing into clinical practice, such as time constraints and insufficient training, and (c) the need for culturally adapted approaches to motivational interviewing tailored to Omani patients. Although participants appreciated the utility of motivational interviewing to improve patient engagement, they reported inconsistent training and limited opportunities to practise the technique in clinical settings.

**Conclusions:**

The study highlights significant gaps in motivational interviewing training and practice within Oman’s residency programmes. It underscores the necessity for comprehensive, structured motivational interviewing curricula that are sensitive to the local context. Enhancing practical training opportunities may improve the integration of motivational interviewing into patient care, particularly in managing chronic diseases and addiction.

Motivational interviewing is a patient-centred communication style that has been defined as ‘a particular way of talking with people about change and growth to strengthen their own motivation and commitment’.^1^ It aims to elicit and strengthen a person’s intrinsic motivation and commitment to change.^2^ Through motivational interviewing, the clinician helps the patient to identify behavioural problems, explore motivation to change, set goals and develop solutions.^3^ As a medical technique motivational interviewing is based on five key principles: adapting to resistance, showing empathy, refraining from arguments, developing discrepancies and promoting self-efficacy.^4^ The literature has identified specific behaviours that are characteristic of motivational interviewing and that could be taught to clinicians,^3^ and these include:

- seeking to understand the patient’s frame of reference, particularly via reflective listening
- expressing acceptance and affirmation
- eliciting and selectively reinforcing the patient’s self-motivational statements – expressions of problem recognition, concern, desire, intention to change and ability to change
- monitoring the patient’s degree of readiness to change and ensuring that resistance is not generated by jumping ahead of the patient
- affirming the patient’s freedom of choice and self-direction.

The prevalence of chronic diseases is rising and the co-occurrence of multiple chronic conditions in one individual is the new norm in healthcare.^5^ ^,^ ^6^ Many behavioural factors that lead to these diseases are modifiable,^7^ and therefore promoting healthy behavioural changes through motivational interviewing is required to reduce the risk of chronic diseases and to improve their management.

In addiction therapy, significant drop-out rates are widely documented.^8^ Therefore, motivational interviewing has been engaged in a short pre-treatment intervention that aims to increase motivation for ongoing care. The literature has also demonstrated that brief motivational interviewing sessions, when given as a stand-alone treatment, could improve alcohol use disorder outcomes compared with those seen in 12-step programmes and in cognitive–behavioural interventions that are more intensive and prolonged.^9^

Globally, the Motivational Interviewing Network of Trainers (MINT) was set up in 1977 with a focus on ameliorating the methods used in motivational interviewing to enhance the calibre and efficacy of counselling and consultations for expert delivery.^10^ Across the board, many motivational interviewing training programmes are delivered to residents and doctors at an institutional level. As a result of these training programmes, resident physicians’ awareness of motivational interviewing, and their skill level, use of motivational interviewing techniques and confidence in employing these correctly have all grown, as have their perceptions of the significance of employing motivational interviewing skills in clinics.^11^ Pre- and post-tests are commonly used to gauge residents’ success^11^ in these training programmes. The literature discusses lengthy motivational interviewing training programmes.^12^ However, few incorporate themselves into short training approaches. The duration of training workshops for motivational interviewing ranges from 2.5 to 12 h, but it is not clear whether longer training programmes might have better outcomes than shorter ones.^13^

Given that most researched motivational interviewing interventions concern behavioural modifications such as exercise, smoking cessation and addiction management, the literature mostly focuses on family medicine and psychiatry (e.g.^14^ ^–^ ^17^). Owing to the benefits of motivational interviewing, many medical schools include it in their teaching of communication skills to future clinicians, enhancing their knowledge and confidence in providing patient counselling.^18^ A study of family medicine resident physicians and faculty showed a significant increase in knowledge of smoking cessation guidelines after a 2.5 h motivational interviewing symposium.^19^

To date, there have been no studies assessing the motivational interviewing skills of family medicine and psychiatry resident physicians in the Sultanate of Oman. Therefore, this qualitative study aims to explore and identify potential gaps in motivational interviewing training and application and to explore how resident physicians learn motivational interviewing techniques and their ability to apply the skills learned. The findings of this study could guide the development and implementation of motivational interviewing in clinics and curricula in residency programmes in the future.

## Research objective

To evaluate motivational interviewing skills, awareness, training and implementation among psychiatry and family medicine resident physicians enrolled in residency training programmes at Oman Medical Specialty Board (OMSB), Muscat, Oman.

## Method

### Study design

This qualitative study was designed to include semi-structured interviews and focus group discussions with psychiatry and family medicine resident physicians at different training levels affiliated to the OMSB in Muscat, Oman.

The OMSB is an autonomous body specialised in training and qualifying physicians in various medical specialties, such as psychiatry, family medicine and internal medicine. The psychiatry training programme is a 5-year programme that aims to graduate general psychiatry specialists with sufficient knowledge and proficiency in its subspecialties of general adult psychiatry, psychotherapy, child and adolescent psychiatry, addiction psychiatry, geriatric psychiatry, forensic psychiatry, rehabilitation psychiatry, consultation-liaison psychiatry and other pertinent subspecialties. The family medicine training programme is a 4-year programme that aims to graduate physicians capable of preventing, identifying, diagnosing, treating and following up common community diseases.

The OMSB’s residency training programmes have embraced a competency-based curriculum model that is extensive and concentrated on continuing education and patient care. The residency programmes that OMSB offers enhance resident physicians’ learning in different settings, such as hospitals, communities, emergency departments, clinics and primary care facilities, with the involvement of many elective options in which they can pursue their unique interests.

At OMSB, psychiatry residents participate in a 1 day workshop focused on motivational interviewing as an integral component of their residency academic activities. Similarly, family medicine residents attend a 1 day smoking cessation workshop where they are introduced to motivational interviewing techniques. Residents from both specialties say that they have the opportunity to discuss motivational interviewing with senior faculty during morning meeting sessions.

In conducting this study, a constructivist perspective was chosen, which is based on the idea that there is no absolute truth that could be discovered, although it will endeavour to comprehend participants’ viewpoints and experiences.

### Study setting

The study was conducted in Muscat at Sultan Qaboos University Hospital (SQUH). This training hospital is affiliated to the OMSB, where psychiatry resident physicians attend their academic sessions. In addition, the OMSB itself was involved, where family medicine resident physicians attend their academic sessions. In both settings, a quiet room was used to conduct the semi-structured one-to-one interviews and focus group discussions (FGDs).

### Study tool

An interview guide was designed in advance. It included an adapted version of the Helpful Responses Questionnaire (HRQ) to assess the resident physicians’ communication skills in delivering motivational interviewing and an adaptation of the Motivational Interviewing Knowledge and Attitudes Test (MIKAT) to assess their knowledge and attitudes. In addition, physicians’ confidence in using motivational interviewing and their perceptions of its importance and the feasibility of implementing it in their practice were measured using Motivational Interviewing Rulers. The predetermined question guide for interviews used in this study is shown in Appendix 1. The semi-structured FGDs adhered to the Consolidated Criteria for Reporting Qualitative Research (COREQ) checklist.

### Study participants

The selection of resident physicians from the two residency programmes aimed at maximum variation. Therefore, junior and senior resident physicians from years 1 to 5 from the psychiatry residency training programme and residents from years 1 to 4 from the family medicine residency training programme were invited.

Inclusion criteria included being currently enrolled in either of the two residency programmes during the study period and having completed at least one clinical rotation.

Exclusion criteria included residents who were on long-term leave during the data collection period or those who declined to participate.

### Data collection

Seven individual interviews and three FGDs with around five resident physicians per group were conducted to accommodate 22 residents from both training programmes. Data collection started on the 1 January 2024 and ended on the 30 April 2024 when saturation was reached, as no new themes were emerging from the interviews and FGDs, indicating that additional data collection was unlikely to yield further conceptual data. The 22 resident physicians who were enrolled in the study were approached after their programme’s academic session. Those who provided verbal consent were sent an email with the written consent form, and then an interview was scheduled with them based on their convenience. The interviews and FGDs were conducted by a female medical student and a female psychiatry resident physician. All interviews and discussions were audio recorded and transcribed verbatim.

### Data analysis

Data were analysed manually using qualitative thematic analysis. The transcriptions were read several times by the second and third authors (T.A.-M. and K.A.-A.) to obtain a sense of the whole data and extract the themes accordingly. The analysis was carried out manually using a qualitative thematic approach at the manifest level. Drawing inspiration from Braun & Clarke’s standard method, the process involved several steps: first, thoroughly reading the interview transcripts; next, highlighting relevant words; then coding these words in context; classifying them; and finally, identifying the themes along with reflective notes.^20^

The data were then coded and organised according to three emerging themes. Each author independently categorised the data and later shared their findings with the entire team, with the final stage of analysis being reviewed by two authors (T.A.-M. and K.A.-.A.).

**Fig. 1:**
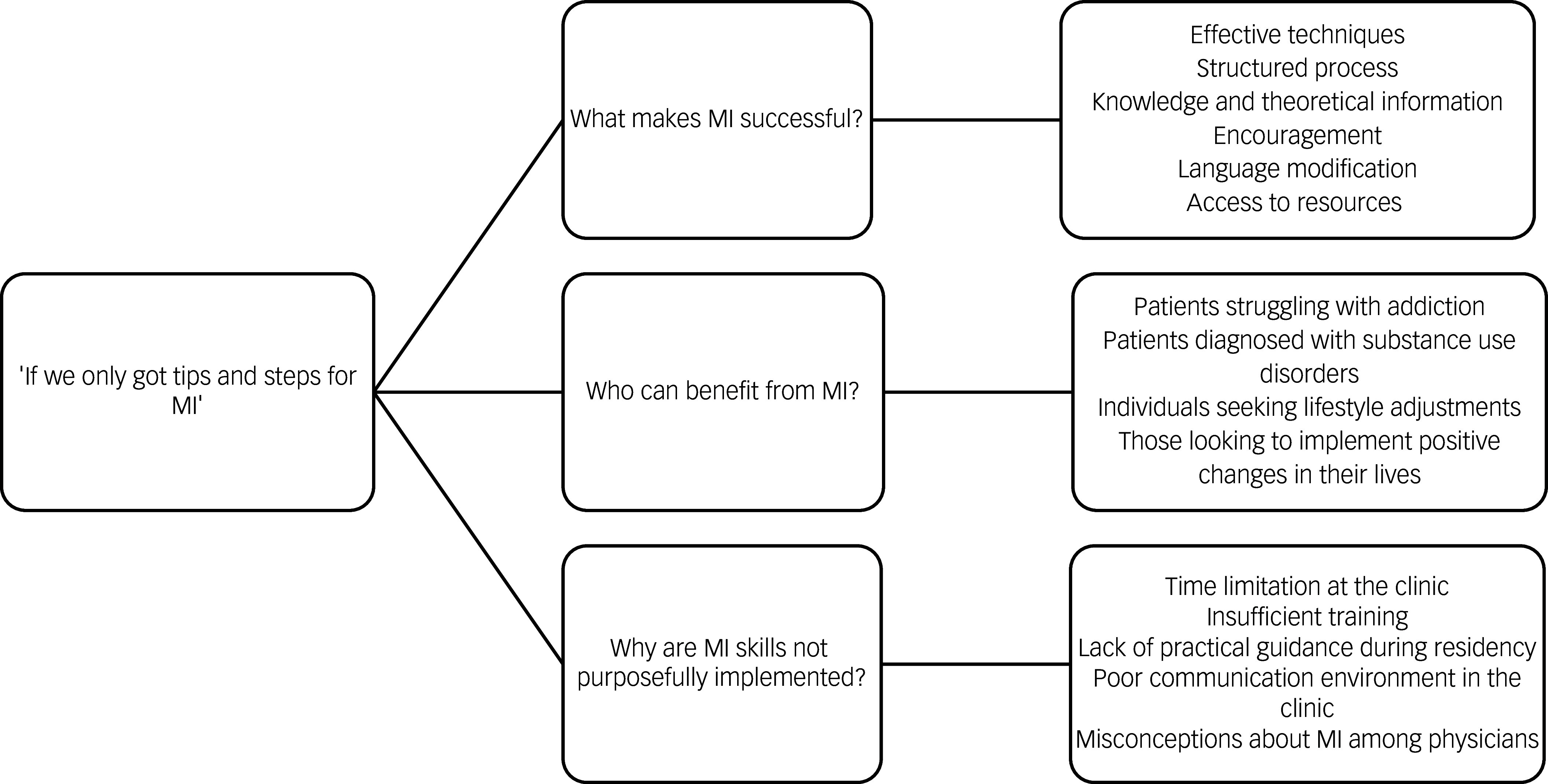
The main theme and sub-themes. MI, motivational interviewing.

### Ethical approval

The study received ethical clearance from the Research and Innovation Committee from OMSB #REC/04/203. The residents were verbally informed about the study with an opportunity to ask the principal investigator any related questions. Participation was established voluntarily, the study objectives along with the procedures were clearly explained and the collected information was handled confidentially. Verbal and written consent were obtained from every resident who agreed to be part of this study. The authors assert that all procedures contributing to this work comply with the ethical standards of the relevant national and institutional committees on human experimentation and with the Helsinki Declaration of 1975, as revised in 2013.

## Results

The analysis resulted in one major theme: ‘If we only got Tips and Steps for Motivational Interviewing’ capturing the positive view of motivational interviewing, along with different challenging aspects. One resident physician admitted the main theme during the interview, providing the importance of its presence in their professional role. Fig. 1 summarises the main findings under three categories. The results below follow these categories and include direct quotations from the dialogues to indicate how the interpretation was grounded in the data.

### What makes motivational interviewing successful?

#### Effective techniques

Discussions with the resident physicians revealed that the majority are well aware of the different motivational interviewing techniques underpinned by key principles, such as expressing empathy, avoiding argument, supporting self-efficacy, rolling with resistance and developing discrepancy. In this aspect, the resident physicians referred to the difference between employing motivational interviewing techniques and traditional counselling practices when it comes to patient outcomes:

‘Motivational interviewing can often be more effective than traditional methods in convincing patients. At times, patients may struggle to accept their treatment plans, and applying motivational techniques can significantly aid in overcoming these challenges’ (Family Medicine Resident Physician, R4).

The resident physicians who had a less comprehensive understanding of the specific techniques that could be employed in motivational interviewing still unanimously agreed on the concept of empathy and its importance as an effective tool. These participants acknowledged that empathetic communication with patients fosters a supportive environment where they feel understood, leading to more positive health outcomes and a greater willingness to participate actively in their treatment plans. They also recognised that even without mastering all the strategies, prioritising empathy can enhance patient engagement and trust, making them feel more empowered:

‘Motivational interviewing may enable us to guide patients more effectively by demonstrating empathy. This approach can lead to greater patient compliance compared to traditional methods that rely primarily on advice. In motivational interviewing, we help patients find their own solutions and encourage them to make changes themselves through empathy and various other techniques’ (Psychiatry Resident Physician, R2).

#### Structured process

One of the challenges that most resident physicians agreed about was their unfamiliarity with the motivational interviewing structure and the process of carrying it out in daily practice. They expressed that they did not feel they understood the structured process adequately to use it in their practice, as well as expressing concerns about their ability to consistently apply these techniques without following the structured process:

‘From my personal experience, I try to motivate the clients who visit my clinic. However, I believe our approach isn’t structured as motivational interviewing. As a result, I sometimes doubt the effectiveness of my efforts in inspiring real change in the clients who receive this motivation’ (Psychiatry Resident Physician, R4).

#### Knowledge and theoretical process

Resident physicians expressed varying familiarity with and understanding of motivational interviewing as a therapeutic technique. Many acknowledged a lack of comprehensive knowledge about motivational interviewing, perceiving it as a technique primarily aimed at addressing specific behavioural changes:

‘We didn’t have much of a comprehensive overview of motivational interviewing. From what I understand, it’s a technique used to motivate patients to overcome specific issues such as obesity, smoking, and other similar challenges’ (Family Medicine Resident Physician, R4).

Furthermore, several resident physicians admitted the need for further education and training in motivational interviewing to account for their lack of knowledge and theoretical underpinnings of the technique:

‘To be honest, I don’t know much about motivational interviewing itself. I definitely need to learn more about it. I think this is true for the majority of our doctors. They don’t have a clear understanding of it. Once they do it, they can provide a better assessment of whether it would be a good approach to implement in practice or no’ (Family Medicine Resident Physician, R3).

It is important to note that despite the lack of familiarity with all the theoretical underpinnings of motivational interviewing, the majority recognised the aspect of empathy:

‘It operates as a stepwise approach, taking into account the Stages of Change: precontemplation, contemplation, action, and maintenance phases. The approach adaptation depends on the patient’s current stage, guiding the process step-by-step from initial engagement through to focusing on the specific changes the patient desires. Ultimately, the final step involves planning – a crucial phase where concrete strategies for change are formulated’ (Psychiatry Resident Physician, R5).

#### Language modification

Resident physicians identified language modification as a critical challenge in implementing motivational interviewing, particularly in the Sultanate of Oman, where there is diversity of culture and languages. This underscores the potential need for using simplified and culturally appropriate language to carry out effective motivational interviewing:

‘In Oman, we have doctors from different nationalities and patients who speak various languages and come from diverse backgrounds. This diversity makes it challenging to use motivational interviewing effectively. We need to adjust how we speak during sessions to ensure patients understand the information while considering their cultural differences and language preferences. That’s why the motivational interviewing approach would need to be adapted considering this point’ (Family Medicine Resident Physician, R2).

Resident physicians also highlighted that sometimes the communication difficulties that some patients have because of their cultural differences are accompanied by other factors, like age and other physical and mental health conditions. Also, some resident physicians mentioned that not all patients are sufficiently cooperative during sessions. These factors make it challenging to effectively apply motivational interviewing, particularly when it comes to using concise and clear communication techniques, and when exploring patients’ perspectives and goals.

‘It’s difficult to understand patient perspectives and thoughts properly. Also, in regards to their treatment, which is a crucial component for motivational interviewing in multiple patients and that could be due to age and various health conditions for example patients with dementia or those with poor insight’ (Psychiatry Resident Physician, R2).

‘Some patients may struggle with communication during motivational interviewing sessions, affecting our ability to guide them effectively’ (Psychiatry Resident Physician, R1).

#### Access to resources

Resident physicians emphasised the importance of access to resources in enhancing their knowledge and confidence to employ motivational interviewing in their practice. One of the suggestions was the utilisation of specialised rooms that are equipped with two-way mirrors when interviewing patients, which could enable resident physicians to be trained efficiently:

‘I’ve learned that some private clinics have rooms with double-sided glass, allowing outside observers to see and hear everything inside. In my opinion, these rooms are ideal for psychiatric interviews, providing comfort for patients and psychiatrists during interactions while allowing supervision. They could be utilised periodically, even with limited resources, for skills evaluation – perhaps bi-weekly or monthly. Initially, training should be provided, followed by ongoing monitoring to track progress in all psychology-related interventions including motivational interviewing’ (Psychiatry Resident Physician, R4).

Regarding educational resources, some resident physicians agreed on the availability of online courses that could be educational, although they were not confident in their reliability:

‘Online courses are increasing widely, but our experience with their suitability for training needs improvement is questionable’ (Family Medicine Resident Physician, R3).

Furthermore, resident physicians stressed the need for comprehensive resources tailored to residents’, needs including self-learning modules, which could help enormously. They also emphasised the importance of accessing materials that support continuous learning and skill development in their practice within their departments:

‘I highly suggest the provision of adequate resources for motivational interviewing training in our department, which would help us build our expertise and develop our skills’ (Psychiatry Resident Physician, R1).

### Who can benefit from motivational interviewing?

Resident physicians underscored motivational interviewing’s benefits and the significant roles these can play when offered to various groups of patients. The technique’s empathetic and non-confrontational approaches help patients explore their uncertainty towards change and eventually increase their readiness to engage in the treatment and recovery process agreed on by the interviewed physicians:

‘The most common use of motivational interviewing in psychiatry is for patients with drug addiction. However, we’re doing it for other patients as well: in general follow-ups, patients who are having medication side effects or others who require treatment plan modification. Also, we apply it to patients who want to improve their lifestyle, exercise, or control their diet. It is part of a holistic care’ (Psychiatry Resident Physician, R5).

The resident physicians highlighted the efficacy of motivational interviewing in patients struggling with addictions and those diagnosed with substance use disorders through repeatedly addressing uncertainty towards recovery, fostering intrinsic motivation and supporting patients in taking active steps towards overcoming addiction:

‘in my current work with addiction cases, I’ve witnessed first-hand the profound effectiveness of motivational interviewing. This approach plays a crucial role in addressing the complexities of addiction, helping individuals navigate ambivalence towards recovery, and empowering them to take proactive steps towards positive change’ (Psychiatry Resident Physician, R4).

Additionally, resident physicians noted that motivational interviewing is highly effective for individuals seeking lifestyle adjustments, whether these involve improving health behaviours, managing chronic conditions or enhancing overall well-being. One chronic condition that has been frequently mentioned as an example is diabetes. Motivational interviewing facilitates discussions about lifestyle modifications such as dietary changes, regular exercise and medication adherence, which are integral to diabetes management:

‘In our practice, we often encounter diabetic patients who resist starting atorvastatin despite guidelines recommending it for managing dyslipidaemia. In such cases, it becomes crucial to engage these patients, explaining the necessity and benefits of atorvastatin to motivate them towards compliance’ (Family Medicine Resident Physician, R4).

### Why are motivational interviewing skills not purposefully implemented?

#### Time limitations at the clinic

One of the primary barriers to the purposeful implementation of motivational interviewing in daily practice was the time limitation in the clinic. Resident physicians highlighted that time constraints during patient consultations often prevent them from engaging in the detailed and reflective conversations required for effective motivational interviewing. The pressure to manage a high number of patients within limited appointment slots of 20 min makes it challenging to allocate the necessary time for motivational interviewing techniques, which are inherently time-consuming:

‘Time constraint is a significant challenge in general practice and specialized clinics, such as diabetes clinics. These settings can be very hectic due to the high volume of patients. As a result, we often do not have enough time to thoroughly explain every aspect of their condition, including treatment protocols, dietary recommendations, and self-care practices at home’ (Family Medicine Resident Physician, R3).

#### Insufficient training

Another significant barrier that was mentioned was the insufficient training for resident physicians in motivational interviewing. Many reported a lack of comprehensive training programmes that adequately cover the principles and techniques of motivational interviewing. Although all the residents in OMSB have access to an online training platform provided by the OMSB called TIBYAN, where they receive medical ethics, communication skills and infection control training, training in motivational interviewing is not yet incorporated into the platform.

Our participants indicated that in the family medicine residency programme, there is a non-mandatory workshop on smoking cessation that addresses several principles of patient motivation. They also reported that some consultants conduct brief discussions with resident physicians during morning meetings. However, properly structured courses in TIBYAN are not available. The participants stated that in the psychiatry residency programme, there is a 1 day theoretical motivational interviewing workshop conducted by a certified motivational interviewer that consists of multiple lectures on what motivational interviewing is and its principles.

The resident physicians feel that there is a gap in motivational interviewing education and training and that this hinders the widespread adoption of motivational interviewing practices in clinical settings:

‘I believe there should be a comprehensive training course that covers motivational interviewing across a broader spectrum, not just for smoking cessation. It should be applied to various diseases and conditions whenever possible’ (Family Medicine Resident Physician, R3).

#### Poor communication and misconceptions about motivational interviewing among physicians

Poor communication and misconceptions about motivational interviewing were other challenges that could be encountered in its purposeful implementation. Some participants implied that they viewed motivational interviewing as a complex or time-consuming technique that does not fit into the fast-paced clinical workflow. Others felt that they may misunderstand and poorly communicate its core principles, leading to scepticism about its efficacy.

These misconceptions can result in a reluctance to adopt motivational interviewing practices, despite their proven benefits in facilitating behaviour change and improving patient outcomes:

‘They don’t believe that it might be effective. They may think it’s overly optimistic or too difficult to apply. Most physicians are very practical, which is why they might not recognize the real power of implementing such an approach’ (Psychiatry Resident Physician, R3).

## Discussion

### The success of motivational interviewing techniques

The family medicine and psychiatry resident physicians in this study perceived motivational interviewing techniques, which included open-ended questions, affirmations, reflective listening and summaries, to be extremely effective in empowering patients and supporting behaviour change compared with traditional advice-giving approaches. These techniques create a foundation for meaningful conversation, allowing physicians to engage patients in a collaborative and non-judgemental manner, therefore promoting patient autonomy and confidence. In addition, the resident physicians described motivational interviewing as a person-centred strategy that enables individuals to strengthen their motivation for change, emphasising positive behavioural outcomes and this is well aligned with the literature.^21^

Some resident physicians, despite their limited understanding of motivational interviewing techniques, agreed on the importance of empathy in creating a supportive environment, leading to positive health outcomes and increased patient participation in treatment plans and adherence, and this is well documented in previous research studies as well.^1^

All the resident physicians in this study acknowledged the effectiveness of motivational interviewing in creating a supportive environment that encourages patients to explore their thoughts, feelings and motivations, ultimately leading to more sustainable behavioural change and improved health outcomes. However, most did not feel confident enough to apply motivational interviewing in clinical practice, either due to limited knowledge or to limited training experience.

The literature on this aspect also supported our results, mentioning that implementing motivational interviewing training into healthcare professions increases physicians’ confidence level in using the technique in clinical settings.^22^

### The target population

Resident physicians highlighted that patients with addictions and those diagnosed with drug use disorders are among the most prominent groups who should receive motivational interviewing. This result is very well aligned with a previous study that showed that motivational interviewing is effective in managing substance use disorder as it addressed hesitancy and conflict, which are considered two of the main characteristics patients with substance misuse could have.^23^

Furthermore, resident physicians stated that motivational interviewing is highly beneficial for those seeking lifestyle adjustments, whether it is to improve health behaviours, manage chronic diseases or improve general well-being. Diabetes is one of the chronic disorders that has been used frequently as an example. Motivational interviewing facilitates discussions about lifestyle modifications such as dietary changes, regular exercise and medication adherence, all of which are essential for diabetes control.^24^

### Implementation challenges

The resident physicians in this study highlighted that a major obstacle to applying motivational interviewing is the language barrier, especially in the Sultanate of Oman, where there is a diversity of cultures and languages. In addition, there are other communication difficulties attributed to age and other mental health conditions. Resident physicians reported that communicating with elderly people who have rigid thinking or mental disorders like dementia or Alzheimer’s could be very demanding. Moreover, the resident physicians stated that not all patients are cooperative during the sessions, which makes motivational interviewing more challenging. Similarly, the literature highlighted uncooperative patients who make motivational interviewing less effective by displaying persistent talk and opposing change, both of which go against the desired behaviour change.^25^

One of the primary barriers to the purposeful implementation of motivational interviewing in daily practice that was mentioned was the time restriction in the clinic. Resident physicians stated that time constraints during patient consultations frequently stop them from engaging in the detailed and reflective conversations necessary for effective motivational interviewing. The need to manage many patients within appointment windows of less than 20 min makes it difficult to devote the necessary time to motivational interviewing methods, which are inherently time-consuming, and this concern is also reported in the literature.^26^ Another significant barrier that was mentioned was residents’ insufficient training in motivational interviewing. Many reported a lack of comprehensive training programmes that adequately cover the principles and techniques of motivational interviewing. This is consistent with previous studies showing that the limited training of physicians in motivational interviewing often fails to equip them with the necessary skills and confidence to effectively use the technique in clinical settings.^22^ The literature continually emphasises the need for more extensive and practical training programmes to improve physicians’ proficiency in the use of motivational interviewing.^22^

These barriers have a substantial impact on the integration of motivational interviewing into clinical practice because they impede the development of proficiency in motivational interviewing techniques owing to limited time for training and practice, leading to a lack of confidence and competence among residents.^1^ In addition, the lack of practical guidance during residency makes it difficult to apply motivational interviewing effectively in real-world patient interactions, and poor environments for communication in clinics prevent the formation of the collaborative and patient-centred care relationships that are required for successful implementation of motivational interviewing.^27^ Addressing these challenges through extensive training programmes, structured guidance and the creation of supportive communication environments is critical to the successful integration of motivational interviewing in clinical practice.

### Cultural context

The study emphasises the necessity for culturally tailored motivational interviewing strategies in Oman; nevertheless, a more thorough examination of cultural elements, including patient–provider interactions and society’s perceptions of behaviour modification, could yield additional insights into the influence of local environment on the adoption of motivational interviewing. In Oman, as in several other nations, cultural norms and traditions significantly influence the relationships between patients and healthcare providers. In certain societies, the provider–patient relationship may be hierarchical, with patients frequently yielding to the physician’s authority. This may pose difficulties in executing motivational interviewing, which depends on a more cooperative, non-authoritarian methodology. Conversely, motivational interviewing’s focus on patient autonomy may encounter opposition in cultures where respect for medical authority is profoundly established.

Moreover, social perceptions of behavioural modification can significantly affect patients’ openness to motivational interviewing. In Oman, as in many regions globally, there may be differing degrees of awareness and acceptance of behavioural health interventions. Social stigma associated with specific health conditions (e.g. addiction, mental illness) may impede patients’ willingness to participate in motivational interviewing, which necessitates transparency and a readiness to examine personal matters. These societal perspectives may establish obstacles that are not readily evident in predominantly Western-centric analyses of motivational interviewing. Consequently, a culturally adapted motivational interviewing strategy that recognises these social dynamics, including the involvement of family members or community leaders when suitable, may improve the efficacy of motivational interviewing interventions.

Integrating cultural competency into motivational interviewing training for healthcare personnel is essential for effective implementation in Oman. Training programmes must have components that enhance understanding of cultural nuances and assist providers in formulating strategies to engage patients in alignment with their values, communication styles and social situations. Comprehending the impact of cultural attitudes on behavioural change would be crucial for enhancing patient engagement in motivational interviewing within Oman’s distinct healthcare context.

### Training and educational resources

The resident physicians in this study emphasised the importance of having available resources to help them improve their knowledge and confidence to employ motivational interviewing in their practice. Some acknowledged that online courses can be instructive in terms of educational materials, but they have doubts regarding their reliability. Others pointed out the importance of providing courses with comprehensive resources, including self-learning modules on motivational interviewing. In addition, they stressed the value of having easily accessible resources through their training programmes that could foster continuous learning and skill development in their practice. These results were also supported by previous studies.^28,29^

The resident physicians believed that poor education and training on motivational interviewing inhibits the wider adoption of motivational interviewing practices in clinical settings. The more wide-spread availability of opportunities already offered in some residency programmes at OMSB, such as the non-mandatory workshop on smoking cessation that covers a few principles on patient motivation, the 1 day workshop on motivational interviewing theory, and consultant-led discussions on the technique during morning meetings with resident physicians, would be a successful move.

Globally, a study in the field of psychiatry declared that more than three-quarters of resident physicians had undergone motivational interviewing education, indicating widespread availability, yet some still believed their interventions were unsuccessful because they had had inadequate motivational interviewing training.^30^ Another survey study conducted in paediatric residency programmes found a lack of formal education in motivational interviewing.^31^ A standardised motivational interviewing curriculum has been shown to increase residents’ confidence in promoting health and behaviour change, which overall highlights the effectiveness of such training programmes.^27^

#### Suggestions for enhancing implementation of motivational interviewing

In the current study, resident physicians suggested implementing continuous workshops from early residency that could provide them with a full understanding of motivational interviewing and allow them to practise the techniques and get ongoing feedback. It was mentioned in the interviews that in Oman, there are only two licensed motivational interviewers, highlighting the need for more licensed individuals in the field of medicine. In addition, as a way of aiding with training, one of the resident physicians raised the possible advantages of using dedicated rooms with two-way mirrors while conducting patient interviews. This would enable the resident physicians to learn practically and receive the needed supervision and feedback through practice and observation, while patients are not distracted by multiple residents and trainers in the consultation room.

Similarly, studies demonstrated that introducing standardised motivational interviewing into the curriculum, such as workshops, review courses and hands-on supervision, can dramatically boost physicians’ confidence in eliciting health behaviour change and utilising motivational interviewing-consistent language.^13^ These findings highlight the importance of enhancing motivational interviewing training resources to better equip physicians with the necessary knowledge and skills to effectively address patients’ ambivalence towards behaviour change.

### Methodological consideration and limitation

The data for this study were gathered by a female medical student and a female psychiatry resident physician. This proved an advantage to the paper as it contributed positively to the need for motivational interviewing in training programmes at OMSB and enhanced the understanding of the experiences of other resident physicians who participated in the study. Moreover, the medical student’s perspective as an outsider was valuable because it allowed an unbiased perception of the residents’ experiences, free from any preconceived assumptions. However, the study had some limitations. Most of the participating residents were female, which may be attributed to the ongoing trend for increasing female representation in Oman’s healthcare workforce across various professions. As a result, no comparison between genders on the aspect of motivational interviewing could be made in this study. It is possible that male residents might experience or express different needs or barriers related to motivational interviewing. Therefore, future studies are encouraged to explore potential gender-based differences to enhance the inclusivity and applicability of motivational interviewing training approaches. Additionally, the study focused on two specialties, which may limit the generalisability of the findings to other residency programmes. However, psychiatry and family medicine are among the most communication-focused specialties in medicine, where motivational interviewing is particularly relevant. Further research involving a broader range of disciplines would nonetheless be beneficial to explore how motivational interviewing training is perceived across diverse medical contexts.

## Data Availability

The data that support the findings of this study are available from the corresponding author, T.A.-M., on reasonable request.

## Appendix 1

### Interview question guide

1. What is your understanding of motivational interviewing?
2. What are your thoughts on the effectiveness of motivational interviewing?
3. How do you think motivational interviewing could be used in your practice?
4. What are the key principles of motivational interviewing?
5. How is motivational interviewing applied in clinical practice?
6. What are the challenges associated with implementing motivational intertwining in clinical practice?
7. How does motivational interviewing work to help patients overcome ambivalence towards behaviour change?
8. How is motivational interviewing different from traditional counselling techniques?
9. What are some common misconceptions or misunderstandings about motivational interviewing?
10. How does motivational interviewing fit into broader treatment programmes for lifestyle modification, substance misuse, mental health and other behavioural health issues?
11. How can motivational interviewing be adapted for use with different populations, such as adolescents or older adults?
12. How can healthcare providers be trained to use motivational interviewing effectively in clinical practice?
13. Have you received any training in motivational interviewing?
14. Do you have any suggestions for improving the motivational interviewing training?
15. Is there anything else you would like to say or add?
