# Motivational interviewing training experiences among psychiatry and family medicine resident physicians: qualitative exploration in Oman – CORRIGENDUM

**DOI:** 10.1192/bji.2025.10060

**Published:** 2026-02

**Authors:** Yamamh Al-Jubori, Tamadhir Al-Mahrouqi, Kamila Al-Alawi, Raghad Almojahed, Rawan Albusaidi, Nazik Tayfor Babiker Ahmed, Mohammed Al Alawi

**Keywords:** Medical education, motivational interviewing, psychiatry education, family medicine education, clinical competence, corrigendum

In the original publication of this article, the co-author Raghad Almojahed’s name was incorrectly given as ‘Raghad Mujahad’.

This has since been updated, and this correction published.
